# Nuclear spindles pave the way to metastasis

**DOI:** 10.18632/oncotarget.23728

**Published:** 2017-12-27

**Authors:** Patrick J. Hensley, Natasha Kyprianou

**Affiliations:** Department of Urology, University of Kentucky, Lexington, KY, USA; Department of Molecular Biochemistry, University of Kentucky, Lexington, KY, USA; Department of Toxicology and Cancer Biology, University of Kentucky, Lexington, KY, USA

**Keywords:** therapeutic targeting, prostate cancer, nuclear Transport, cell invasion, cytoskeleton

Compelling functional studies link nucleolar and spindle associated protein 1 (NUSAP1) with prostate cancer progression to metastasis; the findings highlight a new value of NUSAP1 as a biomarker of tumor recurrence and therapeutic targeting in pre-clinical models of advanced prostate cancer.

Prostate cancer accounted for an estimated 26,120 deaths in the United States in 2016. The majority of these deaths occur in patients with metastatic castration-resistant prostate cancer (CRPC) [1]. The recognized architectural and genomic heterogeneity of prostate tumors and the persistent activation of the androgen receptor (AR) signaling are primarily responsible for treatment failure and clinical relapse [2]. Decision-making regarding the management of localized disease and identification of occult, subclinical metastasis must rely on staging and biomarker validation. Identification of new biomarkers to predict prostate cancer progression to metastasis and molecular signatures to define therapeutic resistance among patients with CRPC is an immediate and critical need in order to eradicate lethal disease.

Nucleolar and spindle associated protein 1 (NUSAP1) has been previously identified as a prognostic biomarker in early stage prostate cancer [3]. In the recent issue of *Oncotarget*, Gordon and colleagues interrogated the role of NUSAP1 in prostate cancer progression, using pre-clinical models and human prostate cancer specimens [4]. Functional studies indicate that NUSAP1 promotes invasive and metastatic properties of prostate tumors, by mechanistically modulating family with sequence similarity 101 member B (FAM101B), a transforming growth factor-beta (TGF-β) signaling effector inducing epithelial-to-mesenchymal transition (EMT) and organizing the actin cytoskeleton, the scaffold for integration of membrane and intracellular functions. The findings are of translational significance enabling new mechanistic insights into the contribution of this nuclear spindle player NUSAP1 to prostate cancer cell invasion, as well as defining its clinical value in predicting prostate cancer metastasis.

NUSAP1 is a microtubule and chromatin-binding protein the serves to bind DNA to the mitotic spindle and facilitate crosslinking of microtubules during mitosis. Its validation as a prostate cancer correlative marker non-withstanding, NUSAP1 gene expression is regulated by a promoter binding site for the transcription factor E2F and negatively regulated by the tumor suppressor RB1 [3, 5]. NUSAP1 has been causally linked to cell proliferation based on its role in the mitotic spindle assembly, but the insightful functional approaches in the current study provide compelling evidence on a new role for NUSAP1 in prostate cancer invasion and metastasis. The study shows that loss of *NUSAP1* decreased migration in androgen-independent prostate cancer cells but had limited effect on the growth dynamics of prostate cancer cells *in vitro* (proliferation, apoptosis). *In vivo* assessment of the burden of visceral and nodal metastases in the PC-3 androgen-independent prostate cancer xenograft model, revealed enhanced metastases consequential to NUSAP1 overexpression, while silencing *NUSAP1* reduced metastasis [4].

Genome wide association data analysis revealed highest NUSAP1 transcripts in human metastatic lesions. Microarray analysis showed a common signature of transcription regulation in genes involved in cancer, cell injury, survival and death associated with NUSAP1 under- or overexpression. Furthermore, validated pathway analysis associated this unique signature with tumor progression and patient survival. FAM101B (RefilinB) acts as a molecular switch in the organization of perinuclear actin during EMT [6]. EMT is recognized as a critical venue for epithelial derived tumors to become invasive and rapidly metastasize, with loss of epithelial cell markers and gain of mesenchymal markers at the invasive front. Significantly enough, FAM101B is upregulated by the master EMT-inducer in the prostate, TGF-β, and loss of FAM101B reversed the EMT phenotype exhibited in NUSAP1 overexpressing prostate cancer cells *in vitro*, pointing to the functional interaction between the two players [4].

**Figure 1:**
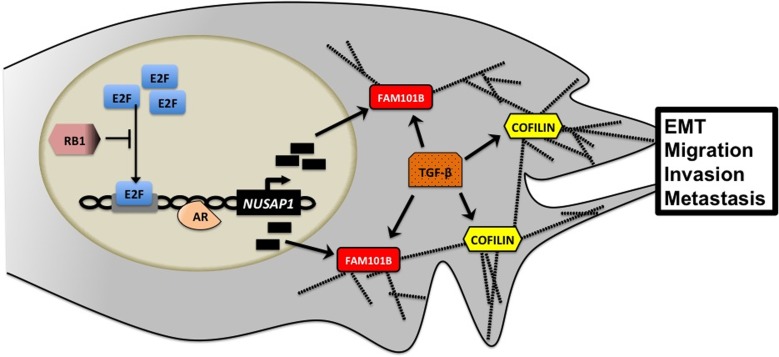
Intracellular NUSAP1 signaling *NUSAP1* expression is promoted by AR and E2F and inhibited by RB1. In the cytosol, NUSAP1 acts under the direction of TGF-β to organize the actin cytoskeleton and drive EMT towards invasion and metastasis of prostate cancer cells. NUSAP1- Nucleolar and spindle associated protein 1, AR- Androgen receptor, RB1- Retinoblastoma 1, E2F- E2 factor, FAM101B- Family with sequence similarity 101 member B, TGF-β- Transforming growth factor-beta, EMT- Epithelial-to-mesenchymal transition

Elucidation of downstream signaling targets/effectors of NUSAP1 is currently under mechanistic pursuit with evidence so far implicating the retinoblastoma RB1 tumor suppressor as a leading candidate, since the transcriptional regulation of NUSAP1 expression is under the control of the well-defined E2F-RB1 pathway (Figure 1). Thus RB1, loss of which correlates with prostate cancer virulence, provides a mechanism bridging upregulation of NUSAP1 to the invasive properties of prostate cancer cells [5]. Further molecular evidence indicating an AR binding site upstream of the NUSAP1 transcription start site and NUSAP1 induction in response to androgens in androgen-sensitive prostate cancer cells [7], positions the AR as a mechanistic protagonist in the upregulation of NUSAP1 in metastatic prostate cancer. Growth stimulation promotes the actin polymerization assembly to generate movement, via cofilin, a TGF-β signaling effector recruited to stabilize perinuclear and cytosolic actin bundles towards an EMT phenotype [8], thus navigating the actin cytoskeleton polymerization in prostate cancer cells (Figure 1). Mutational inactivation of cofilin, significantly compromises prostate cancer cell adhesion, migration and filpodia formation due to enhanced polymerization of F-actin filaments [8].

The role of NUSAP1 in regulating microtubule assembly and stability during mitosis, through its signaling effector FAM101B, provides new target leads. Taxane-chemotherapy is a clinically effective treatment for metastatic CRPC through stabilizing β-tubulin within the microtubule structure, deregulation of the mitotic spindle, apoptosis induction and impairing AR activity [1, 2]. Considering that therapeutic resistance leads to recurrent disease in CRPC patients [2], NUSAP1 presents itself at a convergence targeting point, calling for synergy in action against NUSAP1 in combination with taxane chemotherapy in CRPC. Support for an optimization platform is granted from recent evidence that taxane-induced apoptosis in oral squamous cell carcinoma was enhanced in the absence of NUSAP1 [9].

With the knowledge that development of castration resistance is pivotal in the progression to aggressive disease, it is critical that future investigative efforts focus on CRPC tumors with diverse AR status that may impact the effects of NUSAP1 on the dynamics of microtubules and the actin cytoskeleton towards metastasis. The preclinical models used in this study were limited by their inability to capture and characterize the complexity of the tumor microenvironment during advanced metastatic progression [4]. Utilization of relevant models of prostate cancer metastasis will enable biological outcomes as to the impact of NUSAP1 on the EMT landscapes and microtubule stabilization dynamics, ultimately defining its therapeutic targeting value by combinatorial synergy of FDA approved agents with new drugs (Figure 1). Development of a novel inhibitor targeting NUSAP1 and its nuclear partners, might deliver a personalized therapy with a promise in the clinical management of patients with advanced prostate cancer.
